# The Phenylethanol Glycoside Liposome Inhibits PDGF-Induced HSC Activation via Regulation of the FAK/PI3K/Akt Signaling Pathway

**DOI:** 10.3390/molecules24183282

**Published:** 2019-09-09

**Authors:** Shi-Lei Zhang, Long Ma, Jun Zhao, Shu-Ping You, Xiao-Ting Ma, Xiao-Yan Ye, Tao Liu

**Affiliations:** 1 Department of Toxicology, School of Public Health, Xinjiang Medical University, Xinjiang Uyghur Autonomous Region, Xinyi Road No.393, Urumqi 830011, China (S.-L.Z.) (L.M.) (S.-P.Y.) (X.-T.M.) (X.-Y.Y.); 2 Key Laboratory for Uighur Medicine, Institute of Materia Medica of Xinjiang, Xinjiang Uyghur Autonomous Region, Tianshan District, Xinhua South Road No. 140, Urumqi 830004, China

**Keywords:** phenylethanol glycosides liposome, hepatic stellate cells, proliferation, apotosis, cell cycle, FAK/PI3K/Akt

## Abstract

*Cistanche tubulosa* is a traditional Chinese herbal medicine that is widely used to regulate immunity, and phenylethanol glycosides (CPhGs) are among the primary components responsible for this activity. However, the application of CPhGs is negatively affected by their poor absorption and low oral utilization. Targeted drug delivery is an important development direction for pharmaceutics. Previous studies have indicated that CPhGs could block the conduction of the signaling pathways in TGF-β1/smad and inhibit the activation of hepatic stellate cells (HSCs). The aim of this study was to evaluate the anti-hepatic fibrosis effect of CPhG liposomes by inhibiting HSC activation, promoting apoptosis, blocking the cell cycle, suppressing the conduction of signaling pathways in focal adhesion kinase(FAK)/phosphatidylinositol-3-kinase(PI3K)/protein kinase B(Akt), and determining their in vitro hepatoprotective activity. In vitro release studies demonstrated that CPhG liposomes have a sustained release effect compared to drug CPhGs. HSC proliferation was inhibited after treatment with the CPhG liposomes (29.45, 14.72, 7.36 µg/mL), with IC_50_ values of 42.54 µg/mL in the MTT assay. Different concentrations of the CPhG liposomes could inhibit HSC proliferation, promote apoptosis, and block the cell cycle. The MTT method showed an obvious inhibition of HSC proliferation after CPhG liposome and Recombinant Rat Platelet-derived growth factor-BB(rrPDGF-BB) treatment. The levels of collagen-1, metallopeptidase inhibitor 1 (TIMP-1), α smooth muscle actin (α-SMA), and phosphorylated PI3K/Akt were downregulated, and matrix metalloproteinase-1 (MMP-1) was upregulated, by pretreatment with different concentrations of CPhG liposomes. Moreover, 29.45 μg/mL of CPhG liposomes could decrease the expression of the FAK protein and the phosphorylated PI3K and Akt protein downstream of FAK by overexpression of the FAK gene. This experiment suggests that CPhG liposomes may inhibit the activation of HSCs by inhibiting FAK and then reducing the expression of phosphorylated Akt/PI3K, thereby providing new insights into the application of CPhGs for liver fibrosis.

## 1. Introduction

Liver fibrosis has become an increasing important health-related issue, as it is the wound healing response to various chronic liver injuries, the essence of which is the accumulation of the extracellular matrix (ECM) [1,2]. HSCs are the main cells that generate ECM components in the liver, which must be activated and transformed into myofibroblast-like cells [3]. The stimulation of some pathological factors, such as TGF-β1, alcohol, toxins, and steatosis, can result in the activation of HSCs. The PDGF can also lead to the activation of HSCs [4]. At present, the therapeutic strategies targeting HSCs to prevent liver fibrosis include the inhibition of HSCs activation, proliferation, and cell cycle, as well as the stimulation of HSCs apoptosis [5].

*Cistanche tubulosa* (family Orobanchaceae), a parasitic plant, is widely grown in the southern region of Xinjiang in China. *C. tubulosa* has been shown to present various activities, including enhancing organism immunity, improving organism endurance, nourishing the kidneys, treating impotence, and increasing intelligence; it also has anti-oxidation and anti-aging properties [6]. This plant contains phenylethanol glycosides (CPhGs), iridoids, and polysaccharides, of which CPhGs are some of the main active bioactive species [7]. In a previous study, we found that CPhGs could block the conduction of the signaling pathways in TGF-β1/smad, inhibit the activation of HSCs, and exert preventive effects on bovine serum albumin-induced hepatic fibrosis in rats [7,8]. However, CPhGs have good water solubility and poor lipid solubility and are affected by external factors such as acidity and decomposition enzymes, which reduce the stability and effectiveness of CPhGs [9]. After intragastric administration, CPhGs are unstable in the gastrointestinal tract of rats, and the hydrolysis of glycoside bonds could easily occur. The oral bioavailability of rats was only 0.83%, so it is difficult to determine its therapeutic role [10]. Therefore, we must find a safe, effective, sustained, and targeted antifibrotic drug for HSCs to improve their therapeutic efficacy. Nanoparticle drug delivery systems provide an alternative strategy to overcome these deficiencies because of their advantages, including a high drug loading capacity and long blood circulation [11]. They have a strong affinity to the liver and can directly deliver drugs into cells through endocytosis and fusion. Unmodified liposomes are mainly distributed in the tissues or organs developed by the reticulo-endothelial system (RES) and have the function of passively targeting the liver [12]. Therefore, CPhG liposomes were prepared by membrane dispersion and secondary encapsulation to provide an anti-fibrosis effect. In this study, we aimed to investigate the effects of CPhG liposomes on HSC proliferation, apoptosis, and cell cycle, as well as its mechanisms.

## 2. Results

### 2.1. Physical Properties of CPhG Liposome

**Figure 1:**
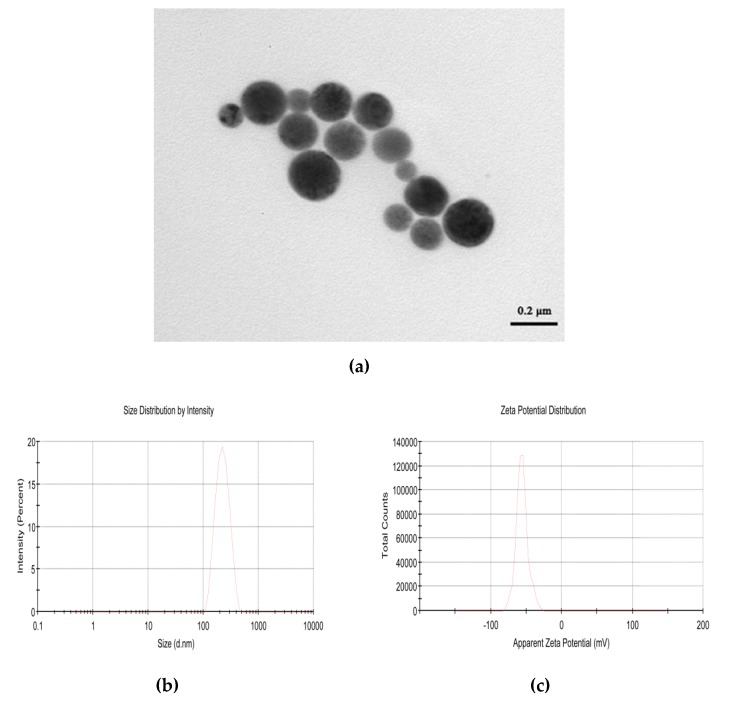
Physical properties of the CPhG liposomes: (**a**) Transmission electron microscopic image of the CPhG liposomes with a scale of 200 nm; (**b**) the laser particle size analyzer particle size distribution profile of the CPhG liposomes; (**c**) potentiometric analysis profile of the CPhG liposomes.

The physical properties of the as–prepared CPhG liposomes were as follows: The particle size was (216.7 ± 3.47) nm, the Zeta potential was (−55.6 ± 1.3) mV. As shown in Figure 1, the particles were round-like, coated with a uniform thickness on the surface, and the particle size distribution was uniform. The drug loading and encapsulation efficiency of the CPhG liposomes were (3.71 ± 0.32)% and (38.46 ± 7.85)%.

### 2.2. In Vitro Release from the CPhG Liposomes and CPhGs

**Figure 2:**
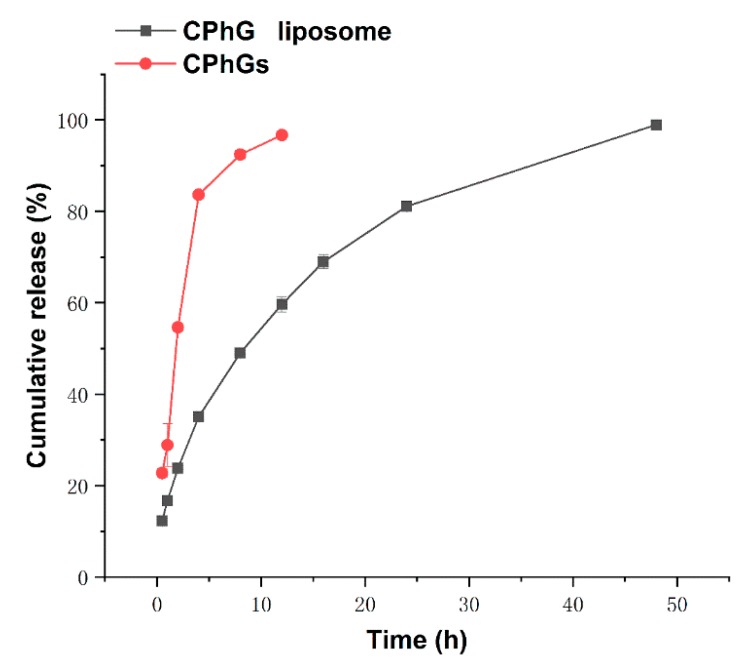
Cumulative release curves of polyphenol levels (%), normalized to the pure extract calculated as the echinacoside equivalents of CPhGs (100% release) for non-encapsulated CPhGs and the CPhG liposome over 48 h.

The estimated cumulative release rate was calculated and plotted as a function of time for the CPhG liposomes and CPhGs (Figure 2). In the present study, the release rate of the CPhGs after 12 h was normalized to 100%, while that of the CPhG liposomes was close to 100% after 24 h, and the release rate of the CPhGs increased with time. Data were analyzed by using different fitting models for the controlled release mechanisms, including the zero-order, first-order, and Higuchi equations. The fitted equations and correlation coefficients for three release models are shown in Table 1.

The best model fits for CPhGs and the CPhG liposomes are the Higuchi and first-order equations. The in vitro release study of the CPhG liposomes did not show any burst effect. The best fitting distribution function was selected to calculate the dissolution parameters T50 (dissolution 50%). The results show that the average retention time of the CPhG liposomes is longer than that of the CPhGs, and the T50 of the CPhG liposomes is 9.39 h. Compared with the 1.69 h for the CPhGs, the CPhG liposomes exhibit an obvious sustained release effect (Table 2).

### 2.3. Cell Proliferation and Toxicity of CPhG Liposomes on HSCs

**Figure 3:**
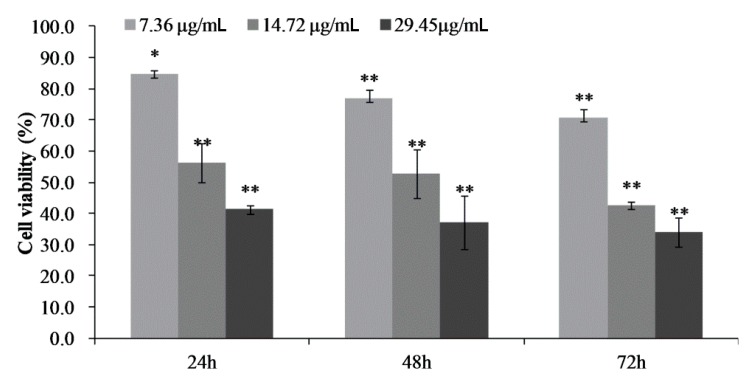
Effects of the CPhG liposome on the activation of HSCs. The cell viability of HSCs after 24, 48, and 72 h of exposure to the CPhG liposome determined by the MTT. Data are presented as the mean ± SD (*n* = 4). * *p* < 0.05 and ** *p* < 0.01 compared with the control groups.

We first investigated the cytotoxic effects of the CPhG liposomes on HSCs with an MTT assay in vitro. HSCs were exposed to different concentrations of the CPhG liposomes, ranging from 0 to 117.79 μg/mL for 24 h. The CPhG liposomes decreased the viability of HSCs in a dose-dependent manner, and their IC_50_ value was 42.535 μg/mL. The effect of the CPhG liposomes (29.45, 14.72, and 7.36 μg/mL) on the cells at 24, 48, and 72 h is shown in Figure 3. After treatment with the CPhG liposomes, the proliferation of HSCs was significantly inhibited at different time points (Figure 3).

The lactate dehydrogenase (LDH) release of HSCs was detected after 24 h of treatment with different concentrations of the CPhG liposome. The results are shown in Table 3. HSCs treated with the CPhG liposomes had no significant effect on LDH release.

### 2.4. CPhG Liposomes Induce HSCs Apoptosis and Arrest the Cell Cycle

**Figure 4:**
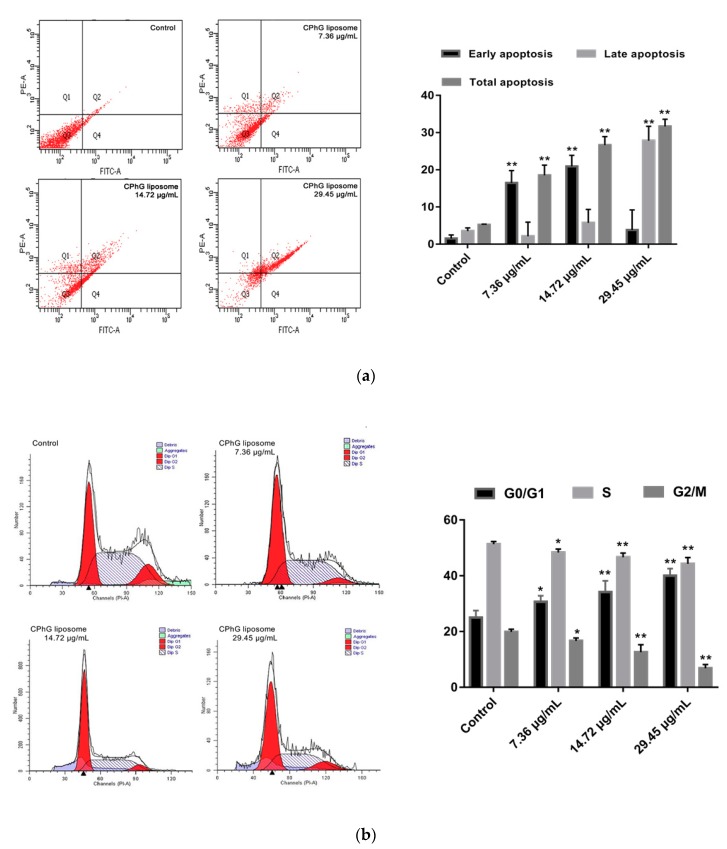
Effect of the CPhG liposomes on the cycle and apoptosis of HSCs. (**a**) Apoptosis of HSCs was analyzed by flow cytometry with AnnexinV-FITC and PE Staining. (**b**) Cell-cycle analysis of HSCs by flow cytometry with PI Staining (*n* = 3). Results are the mean ± SD from three independent experiments. * *p* < 0.05, ** *p* < 0.01 versus the control values.

HSCs were exposed to the CPhG liposomes (29.45, 14.72, 7.36 μg/mL) for 24 h, and annexinV-FITC/PE double staining was carried out to detect apoptosis by flow cytometry, as shown in Figure 4a. The total apoptosis of the cells statistically increased compared to the control group (*p* < 0.05). After 24 h of treatment on HSCs for each dose group, the CPhG liposomes (29.45 μg/mL) could induce late apoptosis of the HSCs, and the percentage of the late apoptotic cells was higher than that of the early apoptotic cells. The proportion of early apoptotic cells was much higher than the late apoptotic cells in the CPhG liposome (14.72 and 7.36 μg/mL)-treated groups. These results clearly indicate that CPhG liposomes induce hepatocyte apoptosis, especially in late stages of HSCs. In addition, total apoptosis increased with the dosage of the CPhG liposomes. These results suggest that CPhG liposomes can inhibit the activation of HSCs and hepatic fibrosis by inducing apoptosis of HSCs.

To determine the effect of the CPhG liposomes on the cell cycle of HSCs, HSCs were treated with the CPhG liposomes at concentrations of 29.45, 14.72, and 7.36 μg/mL for 24 h, and analyzed by flow cytometry (Figure 4b). The results showed that the proportion of cells in the G0/G1 phase was significantly increased (*p* < 0.05), while the percentage of cells in the S phase was significantly reduced (*p* < 0.05). Moreover, the proportion of cells in the G2/M phase was significantly reduced when compared to the control group (*p* < 0.05). Taken together, these results exhibit the cell cycle modulatory activity of the CPhG liposomes in HSCs, which may relate to their anti-proliferative and apoptosis-inducing effects.

### 2.5. Study on the Mechanism(s) of Action of CPhG Liposomes In Vitro

#### 2.5.1. CPhG Liposomes Inhibit HSCs Proliferation by rrPDGF-BB Stimulation

**Figure 5:**
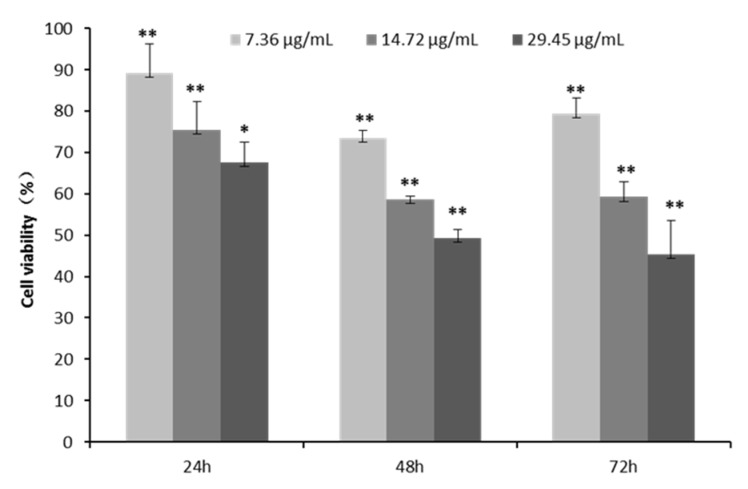
Effect of the CPhG liposomes on HSCs viability. HSCs were pretreated with rrPDGF-BB stimulation before the CPhG liposomes at various concentrations for 24, 48, and 72 h. ** *p* < 0.01, * *p* < 0.05; results were significantly different compared with the rrPDGF-BB-treated HSCs group.

To further investigate the effects of CPhG liposomes on the proliferation stimulated by the rrPDGF-BB of HSCs, an MTT assay was performed to measure the viability of HSCs that were treated with preset concentrations of the CPhG liposomes for 24, 48, and 72 h (Figure 5).

After 24 h of treatment with HSCs stimulated by rrPDGF-BB and 29.45, 14.72, and 7.36 μg/mL of the CPhG liposomes, the survival rates of the cells in each dose group were 67.5%, 75.3%, and 89.2%, respectively. After 48 h, the survival rates were 49.2%, 58.6%, and 73.5%, respectively, in the cells of each dose group. After 72 h of treatment, the survival rates of the cells in each dose group were 45.3%, 59.2%, and 79.2%, respectively. The results showed that the CPhG liposomes had a significant inhibitory effect on HSCs stimulated by rrPDGF-BB, and this inhibitory effect became more obvious as the dose was increased. It is suggested that the inhibitory effect of the CPhG liposomes on HSCs contributes to the CPhG liposome anti-proliferation activity.

#### 2.5.2. The CPhG Liposomes Inhibit HSC Activation In Vitro

**Figure 6:**
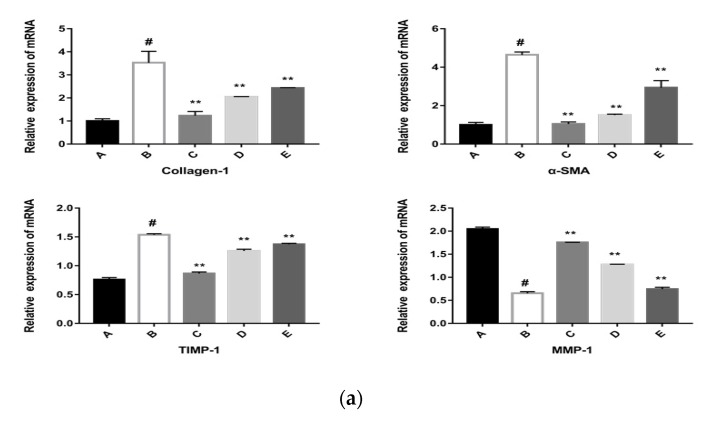

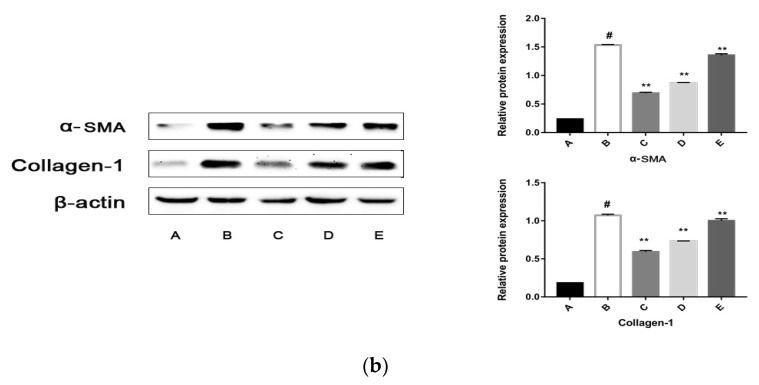
Effects of the CPhG liposomes on the expressions of collagen-1, α-SMA, TIMP-1, and MMP-1 in HSCs. (**a**) The mRNA expression of collagen-1, α-SMA, TIMP-1, and MMP-1 in HSCs (RT-PCR assay). (**b**) The protein expression of collagen-1 and α-SMA. A, the control group; B, the rrPDGF-BB treated group; C, the rrPDGF-BB + CPhG liposome 29.45 μg/mL treated group; D, the rrPDGF-BB + CPhG liposome 14.72 μg/mL treated group; E, the rrPDGF-BB + CPhG liposome 7.36 μg/mL treated group; Data are expressed as the mean ± SD. ** *p* < 0.01, significantly different compared to the rrPDGF-BB-activated HSCs group. # *p* < 0.05, significantly different compared to the control group. β-actin was used as an internal control.

HSCs in the space of Disse produce ECM components like collagen-1, α-SMA, and collagen III. Other factors that regulate the development of ECMs, such as MMPs and TIMPs, are also produced by HSCs [5]. To further investigate the mechanisms of rrPDGF-BB-induced in HSCs, we evaluated the expression of ECM-related proteins, including collagen-1, α-SMA, collagen III, MMP-1, and TIMP-1. Figure 6a demonstrates that the CPhG liposomes (29.45, 14.72, 7.36 μg/mL) groups could reduce the mRNA expression levels of collagen-1, α-SMA, and TIMP-1 and increase the mRNA expression level of MMP-1 more than the rrPDGF-BB group.

The CPhG liposomes also elevated the mRNA levels of MMP-1. The levels of α-SMA and collagen-1 were highly expressed in rrPDGF-BB-activated HSCs. In contrast, the CPhG liposomes decreased the levels of α-SMA and collagen-1 (Figure 6b). Especially at a high concentration (29.45 μg/mL), the CPhG liposome reduced the increased collagen-1 and α-SMA expression via rrPDGF-BB.

#### 2.5.3. CPhG Liposomes Inhibit the PI3K/Akt Signaling Pathway

**Figure 7:**
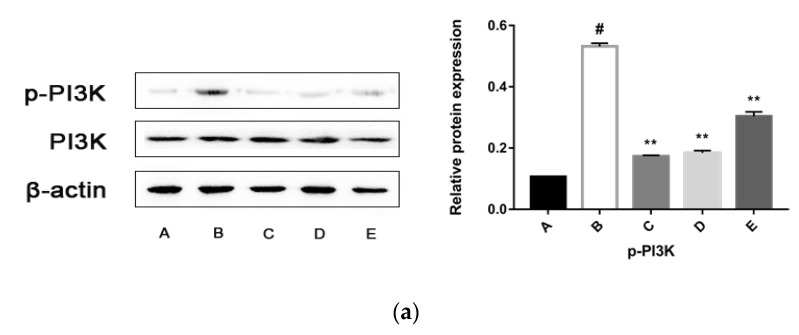

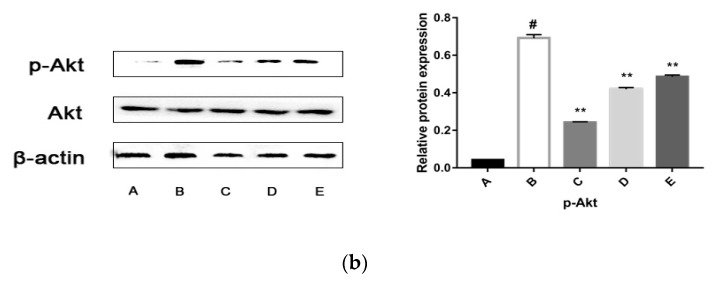
Effects of the CPhG liposomes on the expressions of p-PI3K, PI3K, p-Akt, and Akt in HSCs. (**a**) The protein expression of p-PI3K and PI3K. (**b**) The protein expression of p-Akt and Akt. A, the Control group; B, the rrPDGF-BB treated group; C, the rrPDGF-BB + CPhG liposome 29.45 μg/mL treated group; D, the rrPDGF-BB + CPhG liposomes 14.72 μg/mL treated group; E, the rrPDGF-BB + CPhG liposomes 7.36 μg/mL treated group; Data are expressed as the mean ± SD. ** *p* < 0.01, significantly different compared with the rrPDGF-BB-activated HSC group. # *p* < 0.05, significantly different compared with the control group. β-actin was used as an internal control.

PI3K/Akt is an important signal transduction pathway mediated by tyrosine kinase receptors. The PI3K/Akt signaling pathway acts as a sensor in response to extracellular stimuli and mediates the cellular signals, thereby playing a critical role in cell apoptosis and proliferation by affecting the activity of downstream effector molecules [13]. In order to verify whether the PI3K/Akt pathway takes part in the anti-proliferation effects of the CPhG liposomes on HSCs, the expression and phosphorylation levels of PI3K/Akt were examined by western blot analysis. The results showed that the levels of p-PI3K and p-Akt consistently decreased in HSCs after 24 h treatment with the CPhG liposomes, indicating that the anti-proliferation effects of the CPhG liposomes against HSCs are related to deactivation of the PI3K/Akt pathway, as shown in Figure 7.

#### 2.5.4. The CPhG Liposomes Can Down-Regulate the Expression of PI3K /Akt Pathway Proteins in HSCs of the rrPDGF-BB-Mediated FAK Overexpression Plasmid

FAK is a critical gene that regulates the PI3K/Akt signaling pathway. Activated FAK can phosphorylate PI3K, which further leads to the phosphorylation of Akt. FAK mediates the signal transduction of the tyrosine protein kinase receptor, integrin, and other pathways to cells, which are the junction and hub of several intracellular pathways during HSCs activation. Therefore, we speculated that the CPhG liposomes might suppress the activity of the PI3K/Akt signaling pathway by impairing the activation of FAK. We subsequently confirmed this hypothesis by demonstrating the overexpression of the FAK gene.

**Figure 8:**
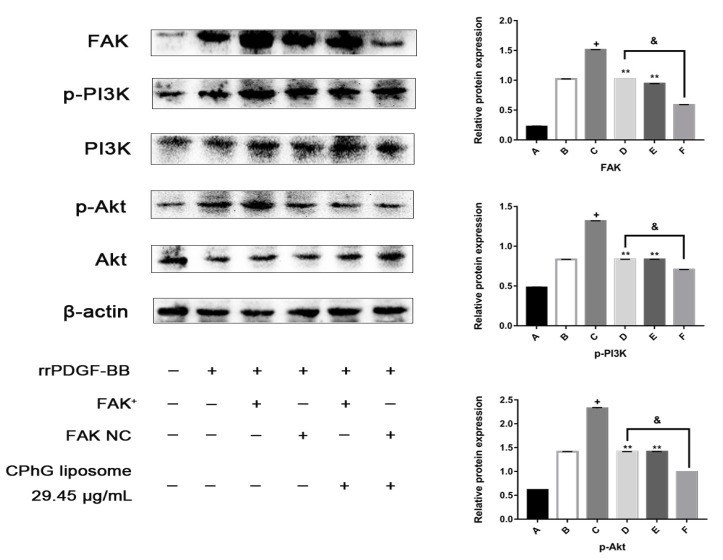
FAK, Phosphorylated and total PI3K and Akt in rrPDGF-BB-activated HSCs after overexpression of FAK. FAK, Phosphorylated and total PI3K and Akt protein were measured by western blotting with specific primary antibodies. ** *p* < 0.01, significantly different compared with the rrPDGF-BB stimulation + pEX-3-FAK group. + *p* < 0.05, significantly different compared with the rrPDGF-BB treated group. & *p* < 0.01, significantly different compared with the rrPDGF-BB stimulation + pEX-3-NC group. β-actin was used as an internal control.

As shown in Figure 8, compared with the rrPDGF-BB stimulation group, the protein expression level of the FAK in the rrPDGF-BB stimulation + FAK overexpression plasmid (pEX-3-FAK) group was significantly elevated (*p* < 0.05), while the expression level of the FAK protein in the rrPDGF-BB stimulation + empty plasmid (pEX-3-NC) group had no significant change. Compared with the rrPDGF-BB stimulation + FAK overexpression plasmid (pEX-3-FAK) group, the rrPDGF-BB stimulation + empty plasmid (pEX-3-NC) group, and the rrPDGF-BB stimulation + FAK overexpression plasmid (pEX-3-FAK) + CPhG liposome 29.45 μg/mL groups could down-regulate the protein expression levels of phosphorylated PI3K and phosphorylated Akt (*p* < 0.01). Compared with the rrPDGF-BB stimulation + empty plasmid (pEX-3-NC) group, the protein expression levels of the phosphorylated PI3K and phosphorylated Akt in the rrPDGF-BB stimulation + empty plasmid (pEX-3-NC) + CPhG liposome 29.45 μg/mL group decreased significantly (*p* < 0.01).

## 3. Discussion

CPhGs have been shown to have a variety of biological activities, including anti-inflammatory activity [14,15], anti-osteoporosis activity [16,17], a sedative effect [18], antifatigue activity [19], a neuroprotective effect [20], and hepatoprotective activity [21,22]. Yang et al. found that an extract of *C. tubulosa* could significantly delay the progress of experimental chemical liver fibrosis by inhibiting collagen synthesis and decreasing oxidative stress [22]. However, the efficacy of CPhGs as hepatoprotectants is limited by their poor permeability and low bioavailability [23]. Therefore, it is necessary to expand the application of CPhGs by developing an efficient delivery system to overcome these setbacks.

In recent years, both drug research and clinical research have confirmed that the use of nanoparticles as drug carriers can improve drug efficacy in vivo. Liposomes are some of the most important nanoparticles [24]. Liposomes can be used as drug carriers to successfully deliver anti-inflammatory drugs, anticancer drugs, antibiotics, antifungal drugs, and other different categories of drugs [25]. Liposomes can also improve drug stability, increase drug solubility, and promote biocompatibility [26]. On the other hand, studies have shown that the drug delivery system of solid nanoparticles (in the range of 10–1000 nm) can concentrate 80% of the drug dosage in the liver, enabling the drug to enter the liver cells [27]. Jing Zhu et al. found that galangin-liposomes possessed good liver targeting effects [28]. Therefore, the passive targeting effect of nano-Chinese medicines can be used to treat liver diseases or other diseases. The particle size of the CPhG liposomes prepared in the present study, which has a certain degree of liver targeting, is (216.7 ± 3.47) nm. Drug release behavior is an important property of nano-drug delivery systems. Because CPhGs have good water solubility, a PBS buffer solution (pH = 6.0) is used as the release medium. From the release curve, it can be seen that before 2 h, the percentage of release of CPhG liposomes was less than 40%, no sudden release occurred, and there was a certain sustained release effect compared with the release of CPhGs. From the fitted release curve equation, the release of the CPhG liposomes in the phosphate buffer solution at pH = 6.0 is consistent with the Higuchi equation.

In chronic liver diseases, the continuous activation of HSCs leads to liver fibrosis [29]. Therefore, inhibiting the activation, proliferation, and cell cycle of HSCs, or inducing HSC apoptosis, may be a new starting point for the targeted therapy of liver fibrosis [30,31,32,33]. Many studies have found that apoptosis is the key to reversing hepatic fibrosis by promoting apoptosis [34]. The development of liver fibrosis depends on the activation of HSCs, which may offer the potential to inhibit HSC activation and induce apoptosis to prevent or treat liver fibrosis [35,36]. LDH is an enzyme in the cell cytosol, which is released in the culture media when the cell membrane is damaged. For these reasons, we performed in vitro studies on the cell viability rates, the content of LDH in cells, and the apoptosis and cell cycle of HSCs exposed to the CPhG liposomes. The results showed that the CPhG liposomes strongly inhibit the viability and proliferation of HSCs and increase the percentage of apoptotic cells in a concentration-dependent manner. Moreover, the results of the cell cycle indicate that the CPhG liposomes could induce cell cycle arrest in the G1 phase. The results show that the key for the CPhG liposomes to prevent and treat hepatic fibrosis is to regulate cell proliferation, apoptosis, and cell cycle. Compared with the blank control group, the LDH content between each group has a gradually increasing trend. However, there was no significant difference (*p* > 0.05), indicating that although the activity decreases for HSCs affected by the CPhG liposomes, most of the cell membranes remain intact. The inhibition of the proliferation of HSCs by the CPhG liposomes is not due to the toxicity of the drugs to the cells.

PDGF is the most critical factors affecting the proliferation of HSCs. The PDGF-receptors (PDGF-Rα and -β) belong to the tyrosine kinase receptor family. PDGF-AA binds exclusively to PDGF-Rα, while PDGF-B chains bind and dimerize with both PDGF-Rα and -β [37]. Following binding of the ligand to the PDGF-R, the receptors dimerize, which subsequently leads to phosphorylation of an internal tyrosine residue and to the activation of several downstream signaling pathways, ultimately inducing the proliferation and migration of activated HSCs. This process results in the excessive production and deposition of collagen and other ECMs, thereby promoting the development and progression of liver fibrosis [38]. Breitkopf et al. showed that the hepatic overexpression of PDGF-BB results in HSC proliferation and liver fibrosis [39]. In our study, we used rrPDGF-BB as a stimulator to activate HSCs, and each dosed CPhG liposome group could inhibit the proliferation of HSCs and present an apparent dose-effect relationship. It is suggested that the inhibitory effect of the CPhG liposomes on HSCs contributes to CPhG liposomes’ anti-proliferation activity.

HSCs activation is associated with many changes in the gene expression patterns of cells. In particular, changes in the gene expression of collagen-1 and α-SMA are most correlated with the fibrosis properties of activated HSCs [40]. MMPs are zinc-dependent endopeptidases that play an important role in the degradation of all ECM protein components under physiological and pathological conditions. The MMPs and TIMPs mediate the synthesis and degradation of the ECM, respectively. MMPs can promote ECM degradation. The delicate balance between MMPs and TIMPs determines the occurrence of liver fibrosis [29]. Compared with the rrPDGF-BB group, the expression of TIMP-1, α-SMA, and collagen-1 in the CPhG liposome groups increased significantly, while the expression of MMP-9 decreased. The CPhG liposome treatment notably reversed the abnormal expressions induced by rrPDGF-BB in HSCs. These data indicate that the CPhG liposomes possibly inhibit the generation of ECM by restoring the balance between MMPs and TIMPs, thereby increasing the levels of MMP-1 and decreasing the expression of TIMP-1, α-SMA, and collagen-1 and potentially promoting extracellular matrix degradation.

FAK is a kind of focal adhesion complex. PDGF can also activate FAK by interacting with the ECM protein through integrin [41]. The focal adhesion complex provides a direct sensor for the integrity of the extracellular environment. The activation of FAK results in the activation of PI3K. PI3K has been shown to be involved in the proliferation of several cell types [42]. Its crucial role in HSCs was shown through the use of PI3K-specific inhibitors LY294002 and wortmannin to block PDGF-induced mitogenesis and chemotaxis without affecting the PDGF receptor autophosphorylation [43]. PI3K is recruited into dimerized phosphorylated PDGF receptors, which activate protein kinase C (PKC), Akt, and p70S6 kinases (p70S6K) [44]. It has been reported that a dominant negative FAK (Ad-FAKCD) was used to block FAK activity and inhibit PI3K activation induced by PDGF and HSCs proliferation after PDGF treatment [45]. Therefore, we hypothesized that the FAK/PI3K/Akt pathway is a possible target for the CPhG liposomes.

Our results demonstrate that compare to the rrPDGF-BB stimulation + pEX-3-FAK group, the protein expression levels of FAK, phosphorylated PI3K, and phosphorylated Akt were downregulated in the rrPDGF-BB stimulation + pEX-3-FAK + CPhG liposome 29.45 μg/mL group. These results show that CPhG liposome 29.45 μg/mL treatment could decrease the expression of the FAK protein and the phosphorylated PI3K and Akt proteins downstream of FAK.

With the deepening of the research on the chemistry and pharmacology of traditional Chinese medicines, more and more active ingredients of traditional Chinese medicines have been found and confirmed. Paclitaxel, artemisinin, and other drugs have been widely recognized as first-line treatment drugs for related diseases around the world. However, at the same time, it is found that although the in vitro pharmacological effect of many Chinese medical ingredients is very strong, they have poor water solubility, a short half-life, poor stability, low bioavailability, toxic side effects, and many other problems that severely limit their medicinal and clinical application. The nano-drug delivery system is a new drug delivery system with great potential for development and is a hotspot in modern pharmaceutics research. Its application in the research and development of new forms of traditional Chinese medicines not only improves the traditional forms of Chinese medicines, but also improves the curative effect of traditional Chinese medicines to a certain extent and increases the stability of drugs in vivo. Moreover, unmodified liposomes are mostly absorbed by the RES, depending on their particle size, surface properties, and other factors. They are also passively targeted to the liver. In order to improve the liver targeting efficiency of ordinary liposomes, the concentration of the target tissue can be increased by connecting specific ligands or specific functional groups.

Therefore, in future research, we should attach importance to the physical and chemical properties of CPhG extraction and purification, such as solubility, stability, acidity, and alkalinity, as well as the study of biopharmaceutics and pharmacokinetics. We should establish a unique formulation design theory, preparation technology platform, and quality evaluation method for CPhGs and also develop a variety of nano-drug-loading systems for CPhGs, to ensure CPhGs are utilized as thoroughly as possible. Making full use of these advantages will be the direction of our future research.

## 4. Materials and Methods

### 4.1. Materials

CPhGs were purchased from Hetian Di Chen Medical Biotechnology Co., Ltd. (Xinjiang, China). The content of the echinacea was more than 35%, and that of the acteoside was more than 16%. Lecithin was purchased from Shanghai Lanji Technology Co., Ltd. (Shanghai, China). 1,2-Dipalmitoyl-sn-glycero-3-phosphocholine (DPPC) and cholesterol were supplied by Avanti Polar Lipid Inc. (Birmingham, AL, USA). An immortalized rat hepatic stellate cell was provided by Shanghai Zhongqiaoxinzhou Biotech (Shanghai, China). Dulbecco′s modified Eagle′s medium (DMEM) was bought from Thermo Fisher Scientific, Inc. (Waltham, MA, USA). Fetal bovine serum (FBS) was purchased from Gibco Life Technologies (Waltham, MA, USA). Penicillin (100 μg/mL) and streptomycin (100 μg/mL) were products of Thermo Fisher Scientific, Inc. MTT [3-(4,5-dimethylthiazol-2-yl)-2,5-diphenyltetrazolium bromide] (5 mg/mL) was purchased from Sigma (Oakville, ON, Canada). LDH activity was assessed by using an LDH assay kit from Beijing Solarbio Science & Technology Co., Ltd. (Beijing, China). The FITC Annexin V Apoptosis Detection Kit I and propidium iodide (PI)/RNase Staining Buffer were all purchased from BD Biosciences (San Diego, CA, USA) and rrPDGF-BB was provided by R&D Systems (Minneapolis, MN, USA). The TRIzol reagent was bought from Thermo Fisher Scientific, Inc. The RevertAid First Strand cDNA Synthesis kit was the product of Thermo Fisher Scientific, Inc. (Vilnius, Lithuania). TB Greentm Premix Ex TaqTM II was purchased from TaKaRam (DaLian, China). Ripa pyrolysis solution was provided by Thermo Fisher Scientific, Inc. Protease inhibitor phenylmethyl sulfonyl fluoride and the Broad-spectrum phosphatase inhibitor mixture were bought from Boster Biological Technology Co., Ltd. (Wuhan, China). Protein bands were visualized by a Pierce™ ECL Western Blotting Substrate, which was provided by Invitrogen (Carlsbad, CA, USA). The horseradish enzyme labeling goat anti-rabbit IgG was purchased from ZhongShan JinQiao Shanghai Co., Ltd. (Shanghai, China). The Super Signal West Femto Trial Kit was supplied by Thermo Fisher Scientific, Inc. Rabbit anti-β-actin, α-SMA, collagen-1, FAK, PI3K, phospho-PI3K, Akt, and the phospho-Akt antibody were all purchased from Beijing Bioss Antibodies Biological Co., Ltd. (Beijing, China). The pEX-3-FAK and pEX-3-NC were purchased from Shanghai GenePharma Co., Ltd. (Shanghai, China). The Lipofectamine 2000 was provided by Invitrogen.

### 4.2. Preparation of the CPhG Liposomes

Lecithin, DPPC, and cholesterol (1:2:2, *m*/*m*/*m*) were dissolved in a mixture of chloroform and methanol (2:1 *v*/*v*), rotated to form a film at 49–50 °C, and CPhGs (250 μg/mL) were added subsequently. The reactive solution was rotated and hydrated continuously, and the phospholipid membrane was transferred into the water to form CPhG liposomes. The encapsulated liposome process was repeated according to the above methods. To obtain a uniform liposome, the encapsulated liposome was extruded 5 times with a 0.45 μm microporous membrane and filtrated 18–20 times with a 0.22 μm membrane. The CPhG liposomes were stored at 4 °C. The particle size distribution was measured by a laser particle size analyzer, the particle shape was observed by transmission electron microscopy, and the drug loading and encapsulation efficiency were determined by HPLC.

### 4.3. In Vitro Release from the CPhG Liposome and CPhGs

The in vitro release profile of the CPhG liposomes and CPhGs was carried out using the dialysis membrane method. A total of 5 mL of each sample was transferred into a dialysis bag with a 14,000 Da molecular weight cut-off. The dialysis bag was immersed into 200 mL of PBS buffer solution (pH = 6.0) vibrated at 37 °C. The release medium (3 mL) was withdrawn and replaced with an isopyknic PBS buffer solution (pH = 6.0) at scheduled time intervals (0.5, 1, 2, 4, 8, 12, 16, 24, and 48 h). The concentrations of the echinacoside in the CPhG liposomes and CPhGs in the release medium were determined by HPLC. The cumulative release (%) of the CPhG liposomes and CPhGs was calculated by using the following Equation (1):

Cumulative release (%) = (C*_n_* × V + C*_n_*_−1_ × V*_n_*_−1_)/W × 100% (1)

where C_n_ is the drug concentration in the release medium at the nth sampling point, V is the volume of the released medium, and W is the total dose.

To better understand the vitro release characteristic of the CPhG liposome and CPhGs, three different models were applied to fit the obtained release data: (a) the zero-order kinetics model, (b) the first-order kinetics model, and (c) the Higuchi model given in Equations (2)–(4):

M_t_/M_0_ = kt (2)

M_t_/M_0_ = 1 − exp^(−kt)^ (3)

M_t_/M_0_ = kt^1/2^ (4)

where M_t_/M_0_ is the fraction of curcumin released from the sample at time t, and k is the release constant of the different models. The correlation coefficient is calculated to illustrate the fitting degree between the release model and the data.

### 4.4. Cytotoxicity

For the HSCs, a density of 1 × 10^5^ cells/mL were plated in 96-well plates and allowed to grow overnight at 37 °C in a 5% CO_2_ incubator. Cell vitality was tested on a wide range of CPhG liposome concentrations (117.79, 58.90, 29.45, 14.72, 7.36, 3.68, 1.84, 0.92, and 0.46 μg/mL) to detect the test cells′ vitality and calculate the IC_50_ values. Then, the cells were incubated at 37 °C for 4 h with 20 μL of MTT (5 mg/mL in PBS solution). The purple MTT-Product product was dissolved in a 150 μL DMSO, and a Multiskan Spectrum Absorbance Reader (Fisher Scientific, Inc., Waltham, MA, USA) was used to measure the absorber at 490 nm. The following formula (Equation (5)) was used to calculate the percentage of cell viability:

Cell viability (%) = (1 − mean absorbency in test wells)/(mean absorbency in control wells) × 100(5)

### 4.5. Cell Toxicity of the CPhG Liposomes on HSCs

Cells were seeded into 6-well plates, and the toxicity of the HSCs was measured using an LDH detection Kit. Cells were plated at a density of 2 × 10^5^ cells/well in 6-well plates at 37 °C in an incubator with 5% CO_2_. After overnight growth, cells were treated with CPhG liposomes (29.45, 14.72, and 7.36 μg/mL). A blank (DEME media) control was maintained simultaneously. After 24 h, the cells were collected by trypsin and centrifugation. Each group of cells was administered strictly in accordance with the kit instructions. The LDH leakage rate of the cell culture fluid was then calculated.

### 4.6. Apoptosis Analysis

Cells were cultured into 6-well plates with a density of 2 × 10^5^ cells/well at 37 °C in an incubator with 5% CO_2_. After overnight growth, cells were treated with CPhG liposomes (29.45, 14.72, and 7.36 μg/mL), and then the apoptosis of HSCs was measured using the FITC Annexin V Apoptosis Detection Kit. The concentrations were based on the IC_50_ determined from the cytotoxicity assay. A blank (DEME media) control was maintained simultaneously. After 24 h, the cells were collected by trypsin and centrifugation and washed with PBS and suspended in a 500 μL binding buffer. Then, the cells were stained with 5 μL Annexin V-FITC and 5 μL PE. Cells were incubated at room temperature for 30 min in the dark, and the apoptotic rate was measured using a FACSCalibur Cytometer (Becton Dickinson, San Jose, CA, US).

### 4.7. Cell Cycle Arrest Analysis

HSCs (2 × 10^5^ cells/2 mL/well) were treated for 24 h with CPhG liposomes at 29.45, 14.72, and 7.36 μg/mL in 6-well plates. A blank (DEME media) control was maintained simultaneously. The cells were harvested and washed with 1 mL of PBS and fixed with 75% ethanol overnight at 4 °C. The cells were then centrifuged and resuspended in 500 μL solution of propidium iodide (PI)/RNase staining buffer at 37 °C for 30 min in the dark. Finally, the cells were measured using a FACSCalibur Cytometer (Becton Dickinson). The proportions of the cells in the G0/G1, S, and G2/M phases of the cell cycle were analyzed by the FACS Diva software (Becton Dickinson).

### 4.8. Effect of the CPhG Liposome on the PDGF Mediated FAK and PI3K/Akt Signaling Pathway

#### 4.8.1. Proliferation Assay

The HSC lines (1 × 10^5^ cells/mL) were plated in 96-well plates and allowed to grow overnight at 37 °C in a 5% CO_2_ incubator. After 24 h of cell culture, the cells were randomly divided into the following five groups: A blank control (cultured in DMEM containing 10% FBS only), rrPDGF-BB (cultured in DMEM containing 50 µg/L rrPDGF-BB), a CPhG liposome intervention (stimulated with medium containing 50 µg/mL rrPDGF-BB 24 h). Then, the cells were treated with 29.45, 14.72, and 7.36 μg/mL of CPhG liposomes. The cells were continuously cultured for 24 h, 48 h, and 72 h in the incubator at 37 °C for 4 h with 20 μL of MTT (5 mg/mL in PBS solution). The purple MTT–formazan product was dissolved in 150 μL of DMSO and estimated by measuring the absorbance at 490 nm in a Multiskan Spectrum Absorbance Reader (Thermo Fisher Scientific, Inc.), and the IC_50_ was calculated. Quadruplicate samples were run for each concentration in four independent experiments. The following formula (Equation (6)) calculated the percentage of cell viability:

Cell viability (%) = (mean absorbency in test wells)/(mean absorbency in control wells) × 100(6)

#### 4.8.2. Quantitative Real-Time Polymerase Chain Reaction (qRT-PCR)

Cells were seeded into 6-well plates with a density of 1 × 10^5^ cells/mL. This grouping method is the same as the previous one. After 24 h, the total RNA was extracted from the cultured cells by a TRIzol reagent. The concentration and purity of the RNA were measured at 260/280 nm using an ultraviolet spectrophotometer according to the standard OD_260_/OD_280_ ratio of 1.8–2.0. The reverse transcription reactions were reversely transcribed from the RNA using the RevertAid First Strand cDNA Synthesis kit. A TB Greentm Premix Ex TaqTM II was used to measure gene expression. The qRT-PCR was performed using an ABI QuantStudio™6 Flex Real-Time PCR system (Applied Biosystems, Foster City, CA, USA). Primer sequences were synthesized by Sangon Biotech (Shanghai, China) (Table 4).

RNA expression levels were quantified with SYBR on a 7500 Real-Time PCR System (Applied Biosystems). The relative expression was normalized to the expression of β-actin using 2^−ΔΔCt^ methods. Each experiment was conducted in triplicate.

#### 4.8.3. Western Blot Analysis

The total proteins extracted from HSCs in different groups were prepared as described previously. Briefly, the similar proteins were separated via 10% sodium dodecyl sulfate-polyacrylamide gel electrophoresis (SDS-PAGE) and then transferred to polyvinylidene fluoride (PVDF) membranes (Roche Diagnostics GmbH, Mannhem, Baden-Wuerttemberg, Germany). Then, the PVDF membranes were blocked in 3% BSA and incubated overnight at 4 °C with the primary antibodies. The next day, HRP-labeled goat anti-rabbit IgG (1:25,000 dilution; 1 h) was added. Protein bands were visualized via a Pierce™ ECL Western Blotting Substrate and imaged using the FluorChem E Imaging System (Protein Simple, San Francisco, CA, USA), which were normalized with β-actin as an internal control.

#### 4.8.4. FAK Overexpression Experiments

The cells were divided into the normal medium control group, the rrPDGF-BB stimulation group, the rrPDGF-BB stimulation + pEX-3-FAK group, the rrPDGF-BB stimulation + pEX-3-NC group, the rrPDGF-BB stimulation + pEX-3-FAK + CPhG liposome group, and the rrPDGF-BB stimulation + pEX-3-NC + CPhG liposome group. The HSC lines (1 × 10^5^ cells/mL) were plated in 6-well plates and allowed to grow overnight at 37 °C in a 5% CO_2_ incubator. On the next day, rrPDGF-BB (cultured in DMEM containing 50 μg/L rrPDGF-BB) cells were added and cultured. On the third day, pEX-3-FAK and pEX-3-NC were diluted in an antibiotic-free medium, mixed well with lipofectamine 2000, and allowed to stand at 37 °C for 20 min, before being added to each well of the cells at 37 °C. After being incubated for 5 h, the medium containing 10% serum was replaced for 48 h, and on the fifth day, the medium was replaced with the concentration of 29.45 μg/mL CPhG liposome for 24 h. Then, each well was collected on the second, third, fifth, and sixth days, and the expressions of the FAK, PI3K, Akt, p-PI3K, and p-Akt gene proteins were detected.

### 4.9. Statistical Analysis

Data were expressed as the means ± standard deviation (SD). The SPSS 16.0 software (IBM, New York, NY, USA) was used for statistical analyses. The significance of the difference was calculated by a one-way ANOVA test and followed by an LSD test. The drug dissolution equation was fitted by Origin 8.5 (OriginLab, Northampton, MA, USA).

## 5. Conclusions

In conclusion, our results demonstrate that CPhG liposomes significantly inhibit HSCs activation, markedly attenuating the development of liver fibrosis by increasing apoptosis and regulating the cell cycle in HSCs. Inhibiting the activation of FAK/PI3K/Akt signaling pathways may be the underlying mechanism by which the CPhG liposomes protect against chronic liver disease associated with fibrosis. These findings provide novel insights into the mechanisms of this new drug formulation as an antifibrogenic candidate for the future treatment of hepatic fibrosis. However, the underlying mechanisms are more complex than what is described here, and our results do not exclude the possible involvement of other devices caused by the CPhG liposomes to treat liver fibrosis.

## Figures and Tables

**Table 1:** The results of fitting equation and coefficients.

	Model	Equation	R
CPhGs liposome	Zero-order kinetics	Q = 1.81t + 26.33	0.82442
	First-order kinetics	Q = 94.67(1 − e^0.09t^)	0.95269
	Higuchi	Q = 14.66t^1/2^ + 5.12	0.97661
CPhGs	Zero-order kinetics	Q = 6.26t + 34.45	0.70876
	First-order kinetics	Q = 97.37(1 − e^0.43t^)	0.98557
	Higuchi	Q = 28.48t^1/2^ + 8.98	0.86156

**Table 2:** The dissolution parameters of CPhGs liposome and CPhGs.

	Model	T50 (h)
CPhGs liposome	Higuchi	9.39139
CPhGs	First-order kinetics	1.6972

**Table 3:** Influence of CPhGs liposomes on LDH secretion in HSCs.

Groups	*n*	LDH (U/10^4^ cell)
Control	9	0.62 ± 0.09
CPhG liposomes 29.45 μg/mL	9	0.67 ± 0.07 *
CPhG liposomes 14.72 μg/mL	9	0.66 ±0.07 *
CPhG liposomes 7.36 μg/mL	9	0.63 ± 0.05 *

Data are expressed as the mean ± SD. * *p* > 0.05, compared with the normal group; there was no significant difference.

**Table 4:** The primer sequences used for real-time PCR assay.

Gene	Forward Primer (5′–3′)	Reverse Primer (5′–3′)
collagen-1	AACGCTATTGCCTGATGGACAGTC	CGCTGAGTCAAGGATGACGAAGAG
α-SMA	GCGTGGCTATTCCTTCGTGACTAC	CCATCAGGCAGTTCGTAGCTCTTC
MMP-1	TGTTCGCCTTCTACAGAGGAGACC	TGTCGGTCCACGTCTCATCCAG
TIMP-1	ATCTCTGGCCTCTGGCATCCTC	CGCTGGTATAAGGTGGTCTCGATG
β-actin	CAACCTTCTTGCAGCTCCTC	CGGTGTCCCTTCTGAGTGTT
